# Comparison of Metabolites and Species Classification of Thirteen Zingiberaceae Spices Based on GC–MS and Multi-Spectral Fusion Technology

**DOI:** 10.3390/foods12203714

**Published:** 2023-10-10

**Authors:** Hui Wen, Tianmei Yang, Weize Yang, Meiquan Yang, Yuanzhong Wang, Jinyu Zhang

**Affiliations:** 1 Medicinal Plants Research Institute, Yunnan Academy of Agricultural Sciences, Kunming 650200, China; hwen2023@126.com (H.W.); ytm@yaas.org.cn (T.Y.); yangweize116@163.com (W.Y.); ymquan2023@126.com (M.Y.); boletus@126.com (Y.W.); 2 School of Agriculture, Yunnan University, Kunming 650504, China

**Keywords:** Zingiberaceae spices, GC–MS, multi-spectral fusion, metabolites

## Abstract

Due to a similar plant morphology in the majority of Zingiberaceae spices, substitution and adulteration frequently take place during the sales process. Therefore, it is important to analyze the metabolites and species classification of different Zingiberaceae spices. This study preliminarily explored the differences in the metabolites in thirteen Zingiberaceae spices through untargeted gas chromatography–mass spectrometry (GC–MS) and combined spectroscopy, establishing models for classifying different Zingiberaceae spices. On one hand, a total of 81 metabolites were successfully identified by GC–MS. Thirty-seven differential metabolites were screened using variable important in projection (VIP ≥ 1). However, the orthogonal partial least squares discriminant analysis (OPLS-DA) model established using GC–MS data only explained about 30% of the variation. On the other hand, the partial least squares discriminant analysis (PLS-DA) models with three spectral data fusion strategies were compared, and their classification accuracy reached 100%. Among them, the mid-level data fusion model based on latent variables had the best performance. This study provides a powerful tool for distinguishing different Zingiberaceae spices and assists in reducing the occurrence of substitution and adulteration phenomena.

## 1. Introduction

Zingerberaceae is a family of Zingiberales in the order monocotyledonous plants. There are about 50 genera and 1500 species in this family, which is primarily found in tropical and subtropical areas such as India, Myanmar, Thailand, China, Malaysia, etc. Its distributed center is South and Southeast Asia [1,2]. There are about 20 genera and 216 species in China, distributed mainly throughout southern China [3]. The common Zingerberaceae fruits in the Chinese market include *Amomum villosum*, *Amomum kravanh*, *Amomum tsaoko*, *Alpinia japonica*, *Alpinia zerumbet*, *Alpinia katsumadai*, *Alpinia oxyphylla*, *Zingiber striolatum*, and so on, which are widely used in traditional medicine and food flavorings. Zingiberaceae plants contain a high amount of essential oils, mainly terpenoids, aldehydes, hydrocarbons, alcohols, ethers, ketones, and other aromatic compounds, which are closely related to the pharmacological activities of aromatic and medicinal plants [4].

Although different Zingiberaceae plants have a similar morphology, their chemical composition and pharmacological effects are greatly different [5]. Peng et al. [2] used gas chromatography–mass spectrometry (GC–MS) to extract and analyze the volatile components of 10 species of flowers of Zingiberaceae plants, and the results showed that the 10 species of flowers contained some of the same volatile compounds but the contents were different, and their pharmacological effects were related to the diversity of volatile compounds in these ten plants. Baharudin et al. [6] also found that the antibacterial activity of the essential oils from the three rhizomes of Zingiberaceae related to the qualitative and quantitative differences in the chemical composition of individual essential oils. Due to their similar plant morphology and being often sold after being processed via drying, peeling, and grinding, Zingiberaceae spices are prone to difficulties in classification and confusion, making it difficult to control their quality. Therefore, comparing the metabolites of different species in Zingiberaceae and classifying them has important practical significance. However, current research on Zingiberaceae plants mainly focuses on the pharmacological activities of their components, such as antibacterial activity, antioxidant activity, insecticidal activity, and so on, while there is relatively little research on the differences in the chemical composition of different Zingiberaceae plants [7,8]. Moreover, most research about the chemical composition of Zingiberaceae plants mainly focuses on flowers, rhizomes, and fresh fruits. There has not been a comprehensive comparative analysis of the metabolic components in the dried fruits of these common Zingiberaceae plants. Therefore, comparing the differences in their metabolites can provide a theoretical basis for the evaluation and control of the quality of Zingiberaceae spices.

Due to the fact that Zingiberaceae spices are basically dried, the methods for the comparison of metabolites and species classification in plants usually include chromatography and spectroscopy [9,10,11]. Gas chromatography–mass spectrometry (GC–MS) and liquid chromatography–mass spectrometry (LC–MS) are the most commonly used methods in chromatography; LC–MS is mainly used for detecting non-volatile substances, while GC–MS is commonly used for detecting volatile substances [12]. Currently, there are some previous studies on metabolite identification and species classification based on chromatography, which was mainly used for *Lippia* [13], coffee [14], banana [15], cinnamon [16], and so on. The main active ingredient in Zingiberaceae spices is essential oil, which is mainly composed of volatile substances. Therefore, the method used in most studies on the chemical composition of Zingiberaceae spices is GC–MS [7,8,17]. Spectroscopy is widely used in food quality certification, including metabolite analysis, geographic traceability, and adulteration detection. Pauzi [18] evaluated the ability and effectiveness of Fourier transform infrared spectroscopy (FTIR) and e-nose-GC combined with chemometric techniques to discriminate nine powdered rhizomes of Zingiberaceae in Malaysia. Wahyuni et al. [19] applied proton nuclear magnetic resonance (^1^H-NMR) to analyze the metabolites of three species of *Curcuma*, and the results showed that their primary and secondary metabolites could significantly distinguish the three species of *Curcuma*. Therefore, spectroscopy has certain feasibility and effectiveness in species identification and classification applications.

However, both methods have their advantages and disadvantages; GC–MS has the advantages of high-throughput analysis of a large number of metabolites and can be used for quantitative evaluation, but it has drawbacks such as complex operation, strong intra-class variability, and difficulty in separation and clustering. Spectroscopy has advantages such as speed, simplicity, non-destructive testing, and strong repeatability, but cannot be associated with individual chemical substances [20]. The combination of these two technologies can make up for the shortcomings and combine the advantages, to provide a more comprehensive identification and classification of differential metabolites between different species. Most of the research on the metabolites of Zingiberaceae plants used a single technology. A few studies, such as Qin et al. [21], used GC–MS and near-infrared (NIR) spectra to compare and identify the metabolites of *Amomum tsaoko* and *Amomum paratsaoko*. However, there has been no comprehensive comparative analysis of various common Zingiberaceae spices by combining GC–MS with spectroscopy.

Therefore, the main purpose of this study was to conduct a comprehensive quantitative and qualitative evaluation of thirteen common Zingiberaceae spices in the Chinese market based on GC–MS and spectroscopy. The specific aims were to: (1) compare the differential metabolites of thirteen Zingiberaceae spices using non-targeted GC–MS metabolomics and quantitatively evaluate their key metabolites; (2) qualitative evaluation of thirteen Zingiberaceae spices using near-infrared (NIR) and mid-infrared (MIR) combined spectroscopy, rapid identification, and classification of different Zingiberaceae spices. This study provides a powerful tool for distinguishing different Zingiberaceae spices and helps to reduce the phenomenon of adulteration and mixing of Zingiberaceae spices.

## 2. Materials and Methods

### 2.1. Plant Materials

The dry fruits of thirteen Zingiberaceae [*Amomum villosum* (specimen number: IMDY0023076), *Amomum longiligulare* (specimen number: IMDY0023008), *Amomum kravanh* (specimen number: HITBC0024349), *Amomum tsaoko* (specimen number: HITBC048589), *Amomum koenigii* (specimen number: IMDY0023002), *Amomum paratsaoko* (specimen number: KUN424127), *Amomum maximum* (specimen number: HITBC0023562), *Alpinia japonica* (specimen number: PE02018352), *Alpinia zerumbet* (specimen number: IBK00137266), *Alpinia galanga* (specimen number: IBK00137085), *Alpinia katsumadai* (specimen number: IBK00137278), *Alpinia oxyphylla* (specimen number: IBK00137178), and *Zingiber striolatum* (specimen number: IBK00137750)] were purchased from a local market in China. These thirteen Zingiberaceae spices are the most commonly planted and used in Yunnan, China, and their dried fruits are very similar, leading to frequent adulteration and mixing. All the samples were identified by Dr. Jinyu Zhang (Medicinal Plants Research Institute, Yunnan Academy of Agricultural Sciences, Kunming, China).

### 2.2. Sample Preprocessing for Spectra Acquisition

Each species was purchased in different batches from different suppliers, which is representative and sufficient. Each species of Zingiberaceae was randomly divided into 15 samples, to a total of 195 samples. Each sample was ground to powder and passed through a 100-mesh sieve, and the sifted powders were stored in sealed bags for spectra acquisition. 

### 2.3. Near-Infrared (NIR) Spectra Acquisition

The powder samples were placed on a near-infrared spectrometer (Thermo Fisher Scientific Inc., Waltham, MA, USA) for near-infrared spectra acquisition. Each sample was repeatedly collected 3 times, and the average spectrum was taken for analysis. The conditions for collecting near-infrared spectra were as follows: each sample was scanned 64 times, the spectral resolution was 8 cm^−1^, and the scanning range was 12,000 cm^−1^–4000 cm^−1^.

### 2.4. Mid-Infrared (MIR) Spectra Acquisition

The mid-infrared spectroscopy analysis of the samples was carried out using an attenuated total reflection-Fourier transform infrared spectroscopy spectrometer equipped with a deuterated triglycine sulfate detector (Perkin-Elmer company, Waltham, MA, USA). The crystal material of the attenuated total reflection attachment was diamond. Each sample was scanned 64 times, the spectral resolution was set as 16 cm^−1^, and the scan range was set as 4000–400 cm^−1^. Each sample was collected repeatedly 3 times, and the average spectrum was taken for analysis. 

### 2.5. Gas Chromatography–Mass Spectrometry (GC–MS) Conditions and Measurement

Solid phase microextraction (SPME) technology was used for pretreatment, and a specialized CTC trinity automatic sampler with an extraction head (50/30 μm DVB/CAR on PDMS) was used to extract metabolites from the sample. Helium gas was used with a constant flow rate of 1 mL/min to separate derivatization substances and 5.0 g of sample was added to a 20 mL headspace bottle, sealed with a cap [22]. The internal standard substance used in this experiment was 1,2-dichlorobenzene, at a concentration of 100 μg/mL. The sample was shaken at 250 rpm for 15 min before extraction for 30 min, and the extraction temperature was set to 50 °C. The SPME extraction head was inserted into the upper part of the headspace bottle through the bottle cap. The extraction head was about 1.0 cm above the surface of the sample, and the headspace extraction was carried out for 30 min. After sampling was completed, the extraction head was inserted into the GC–MS injection port for desorption for 5 min and GC–MS analysis was performed. The GC cycle time was 50 min. GC–MS was performed by the 7890B gas chromatograph (Agilent Technologies Inc., Santa Clara, CA, USA) and the Pegasus BT gas chromatography time-of-flight mass spectrometer (LECO, St. Joseph, MI, USA). The chromatographic column was a DB-WAX (30 m × 0.25 mm × 0.25 µm). The temperature program was as follows: the sample inlet temperature was 260 °C, the temperature was set to 40 °C as the initial temperature and held for 5 min, heated up to 220 °C at 5 °C/min and heated up to 250 °C at 20 °C/min and held for 2.5 min, the interface temperature was set as 260 °C, the ion source temperature was 230 °C, the quadrupole temperature was 150 °C. The electron ionization (EI) mode was set as 70 ev, and the quality range of full scan mode was 20–400 m/z. Each Zingiberaceae spice was repeatedly collected 3 times.

### 2.6. GC–MS Data Analysis

The GC–MS data were compared with the standard mass spectrometry database (National Institute of Standards and Technology Library 2014) and the published literature to obtain compound information, retention time, and peak area. To enable comparison of data of different orders of magnitude, internal standard normalization of peak areas was performed on the data. The relative content of each metabolite using the concentration and peak area of the internal standard substance was calculated.

### 2.7. Multivariate Statistical Analysis for Spectra Data

Each class of spectral data was divided into about 70% calibration set and 30% validation set using the Kennard–Stone (KS) algorithm, with 143 samples assigned to the calibration set and 52 samples assigned to the validation set for each class of data. The calibration set was used to establish and optimize the model, and the validation set served as an external validation to evaluate the model’s classification performance. This study used partial least squares discriminant analysis (PLS-DA) algorithms to establish classification models. The supervised classification models for PLS-DA were established using SIMCA 14.1.

## 3. Results and Discussion

### 3.1. Analysis of Metabolites

**Figure 1:**
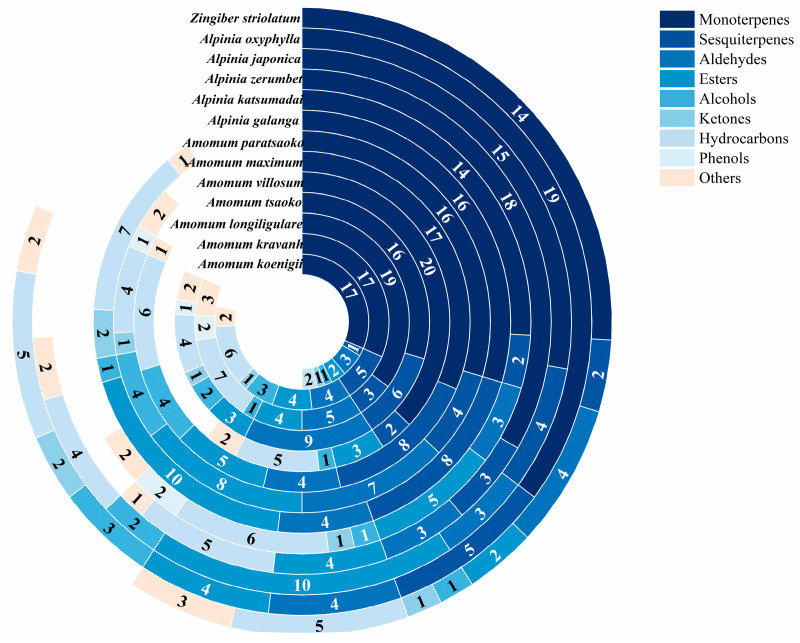
The compound classification diagram of thirteen Zingiberaceae spices.

The volatile components of the thirteen Zingiberaceae fruits were determined by GC–MS, and a total of 81 metabolites were successfully identified, with 27, 42, 44, 44, 33, 45, 47, 49, 36, 34, 40, 44, and 32 volatile metabolites obtained, respectively. They included a wide variety of terpenoids, aldehydes, esters, alcohols, ketones, hydrocarbons, phenols, and other metabolites (Figure 1). Among them, terpenoids had the highest proportion in all samples and most terpenoids were monoterpenoids. It can be seen that the proportion of terpenoids in the detected volatile compounds in different samples ranged from 42.55% to 66.67%. It indicates that terpenoids play an important role in Zingiberaceae spices.

The main volatile components of the thirteen Zingiberaceae fruits are shown in Table 1. It can be seen that the main components of these thirteen Zingiberaceae spices are mostly terpenoids. The metabolite with the highest relative content in *Amomum koenigii*, *Amomum villosum,* and *Zingiber striolatum* was linalool. 1,8-cineole was the metabolite with the highest relative content in *Amomum kravanh*, *Amomum tsaoko*, *Alpinia zerumbet*, and *Alpinia japonica*. In addition, borneol, (–)-β-pinene, (Z)-2-decenal, caryophyllene, estragole, and citral were detected as the metabolites with the highest relative content in each sample from *Amomum longiligulare*, *Amomum maximum*, *Amomum paratsaoko*, *Alpinia galanga*, *Alpinia katsumadai*, and *Alpinia oxyphylla*, respectively. The results indicate that Zingiberaceae plants have significant differences in their relative content; this is also an important reason why they have different biological activities. 

The existing research has shown that the main components of the *Amomum kravanh* fruit are 1,8-cineole, α-pinene, α-terpinene, and β-pinane [23]. The main chemical components of the *Amomum longiligulare* fruit are (–)-bornyl acetate, D-camphor, (E)-nerolidol, camphor, and D-limonene [24]. The main components of *Amomum tsaoko* are 1,8-cineole, β-pinene, α-phellandrene, α-terpineol, geraniol, and geranial [25]. The main components of *Amomum villosum* are camphor, bornyl acetate, β-laurene, α-phellandrene, limonene, terpinene, and linalool [26]. The main components of *Amomum maximum* are β-pinene, β-caryophyllene, α-pinene, sylvestrene, and δ-cadinene [27]. The main components of *Alpinia galanga* are 1,8-cineole, α-fenchyl acetate, β-myrcene, β-ocimene, camphor, and limonene [28]. The main components of *Alpinia zerumbet* are 1,8-cineole, camphor, p-cymene, and terpinen-4-ol [29]. The main components of *Alpinia oxyphylla* are p-cymine, estragol, copaene, and so on [30]. However, there is currently limited research on the composition of the fruits of *Alpinia Katsumadai*, *Amomum paratsaoko*, *Alpinia japonica*, and *Zingiber striolatum*. There were certain differences in the relative content of some compounds from previous reports compared to the results of this study. The reason for this situation was that there were certain differences in the components obtained using different extraction methods. Another important reason was the heterogeneity of the biological samples. It can also be seen that there are certain differences in the metabolites and relative contents detected between the same species. This is because the differences in metabolites are not only related to species, but also influenced by factors such as cultivation conditions, harvesting periods, processing methods, and so on [31]. 

Moreover, the four terpenoids jointly identified from thirteen samples were 1,8-cineole, linalool, α-pinene, and camphene, which have unique functions and biological activities (Table S1). 1,8-cineole is a saturated monoterpene that has the ability to treat respiratory diseases, cardiovascular illness, and digestive sickness [32]. As a kind of acyclic monoterpene alcohol, linalool naturally presents in various aromatic plants and has some bioactive properties including anticancer, antimicrobial, neuroprotective, anxiolytic, antidepressant, anti-stress, hepatoprotective, renal protective, and lung protective activities [33]. α-pinene is a member of the monoterpene group which has been used in the treatment of respiratory tract infections and has antibacterial and insecticidal activities. In addition, it also plays an important role in the flavor industry [34]. Camphene belongs to the group of monoterpene hydrocarbons, and it has been proven that it has antibacterial, anticancer, antioxidant, antiparasitic, antidiabetic, anti-inflammatory, and hypolipidemic activities [35]. Even though the components of the different species have similar metabolic components, there are still certain differences in their relative content.

**Figure 2:**
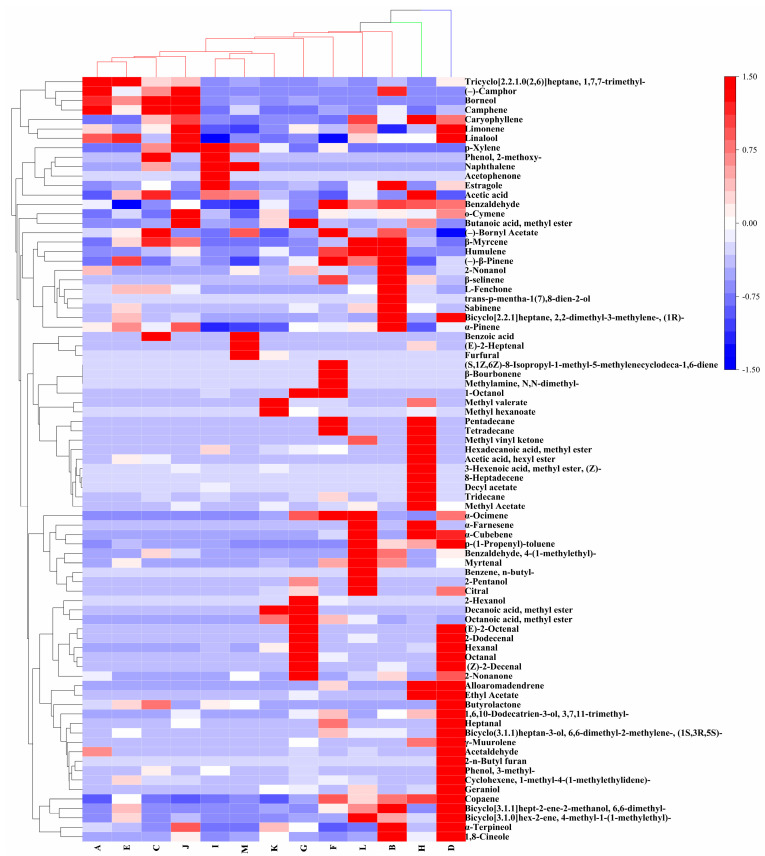
The heatmap of hierarchical clustering analysis (HCA) of thirteen Zingiberaceae spices. A: *Amomum koenigii*; B: *Amomum kravanh*; C: *Amomum longiligulare*; D: *Amomum tsaoko*; E: *Amomum villosum*; F: *Amomum maximum*; G: *Amomum paratsaoko*; H: *Alpinia galanga*; I: *Alpinia katsumadai*; J: *Alpinia zerumbet*; K: *Alpinia japonica*; L: *Alpinia oxyphylla*; M: *Zingiber striolatum*.

Hierarchical clustering analysis (HCA) could more intuitively reflect the difference in the content of metabolites between samples, and the heatmap could further visualize this difference. HCA was performed on metabolites from different samples, and the heatmap is shown in Figure 2. Among them, 9 metabolites, 26 metabolites, 18 metabolites, 36 metabolites, 20 metabolites, 23 metabolites, 18 metabolites, 25 metabolites, 8 metabolites, 17 metabolites, 10 metabolites, 25 metabolites, and 10 metabolites were upregulated in *Amomum koenigii* (A), *Amomum kravanh* (B), *Amomum longiligulare* (C), *Amomum tsaoko* (D), *Amomum villosum* (E), *Amomum maximum* (F), *Amomum paratsaoko* (G), *Alpinia galanga* (H), *Alpinia katsumadai* (I), *Alpinia zerumbet* (J), *Alpinia japonica* (K), *Alpinia oxyphylla* (L), and *Zingiber striolatum* (M), respectively. Among them, *Amomum koenigii*, *Amomum villosum*, *Amomum longiligulare*, and *Alpinia zerumbet* are clustered together due to similar metabolites. Trichyclo [2.2.1.0 (2,6)] heptane, 1,7,7-trimethyl-, (–)-camphor, borneol, and camphene have relatively high levels of metabolites compared to other spices, which is an important basis for distinguishing other spices. Some spices have detected unique metabolites, such as trans-p-mentha-1(7),8-dien-2-ol (*Amomum kravanh*); 2-n-butyl furan (*Amomum tsaoko*); methylamine, N,N-dimethyl-, β-bourbonene, and (S,1Z,6Z)-8-isopropyl-1-methyl-5-methylenecyclodeca-1,6-diene (*Amomum maximum*); 8-heptadecene (*Alpinia galanga*); acetophenone (*Alpinia katsumadai*); benzene and n-butyl- (*Alpinia oxyphylla*). From this, it can be seen that there are similar compounds in these thirteen spices, but each spice has some unique metabolites, so we can distinguish them by these differential metabolites.

**Figure 3:**
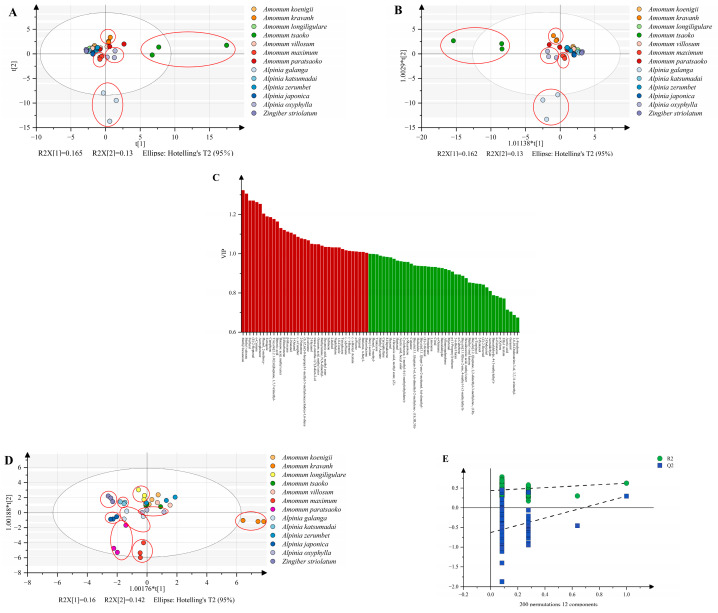
Principal component analysis (PCA) score plot (**A**) and orthogonal partial least squares discriminant analysis (OPLS-DA) score plot (**B**) of gas chromatography–mass spectrometry (GC–MS) from the thirteen Zingiberaceae spices; variable importance for the projection (VIP) of each variable (**C**); OPLS-DA score plot for VIP ≥ 1 (**D**); permutation plot at 200 times of permutations (**E**).

Afterward, multivariate statistical analysis was performed on the GC–MS data, including principal component analysis (PCA) and orthogonal partial least squares discriminant analysis (OPLS-DA), as shown in Figure 3. From the score plots of PCA (Figure 3A) and OPLS-DA (Figure 3B), it could be seen that some species exhibit a clear separation trend, including *Amomum tsaoko*, *Alpinia galanga, Amomum kravanh*, *Amomum maximum*, and *Alpinia oxyphylla* (as shown in the red circle in the figure). The four spices distinguished by PCA and OPLS-DA scatter plots are exactly the four spices with the farthest clustering distance in the clustering heatmap, indicating that the metabolites detected by GC–MS can distinguish and classify different species of Zingiberaceae to a certain extent. However, there was no significant separation between the other spices because the metabolites of these species were relatively similar, as shown in the heatmap, which made it difficult to distinguish them through GC–MS. 

The variable importance in projection (VIP) is the variable weight value of the OPLS-DA model, which can be used to measure the impact and explanatory power of the accumulation differences of various metabolites on the classification and discrimination of each group of samples [36]. VIP ≥ 1 is a common screening standard for differential metabolites, and a total of 37 significant variables were screened (Figure 3C). Among them, terpenoids accounted for 37.84%, including 11 monoterpenes and 3 sesquiterpenes. Monoterpenes and sesquiterpenes are common chemical constituents in Zingiberaceae, and their accumulation differences contribute greatly to the classification and discrimination of samples. The second highest contributor to the model was esters, with methyl acetate having the highest VIP value and having the greatest impact on sample classification. By screening the differential metabolites of different species, the interference of redundant information can be reduced, providing the performance of classification of the model. Therefore, the metabolites with VIP ≥ 1 were screened out for OPLS-DA analysis again (Figure 3D). It can be found that there is an obvious separation trend between more species, and the classification performance of the model is better than the original data model. The OPLS-DA resulted in R^2^(X) of 0.874, R^2^(Y) of 0.84, and Q^2^ of 0.644 of the variables, which indicated good prediction ability of the model. In addition, the results of the permutation testing (n = 200) of the OPLS-DA model show that the model had no risk of overfitting (intercepts: R^2^ = 0.437, Q^2^ = −0.631) (Figure 3E). However, the PCA score plot and OPLS-DA score plot established by raw GC–MS data explained 29.5% (PC1:0.165, PC2:0.13) and 29.2% (PC1:0.162, PC2:0.13) of the variation, respectively. Further, the OPLS-DA score plot established by differential metabolites explained 30.2% of the variation (PC1:0.16, PC2:0.142). It can be observed that the contribution rates of these models are all low, resulting in poor explanatory power of the models. Therefore, it is necessary to combine it with other technical methods (such as spectroscopy) to classify different species, in order to achieve more comprehensive and accurate classification results.

### 3.2. Spectroscopic Analysis

**Figure 4:**
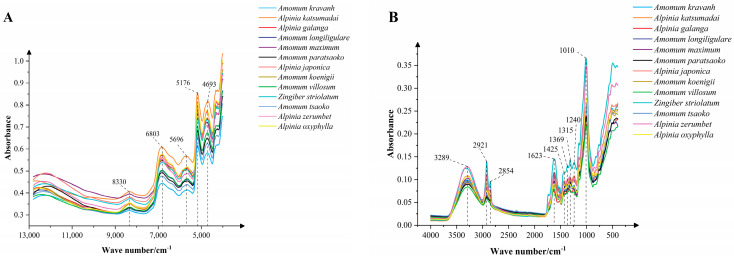
The average near-infrared (NIR) spectra (**A**) and mid-infrared (MIR) spectra (**B**) of thirteen different Zingiberaceae spices.

The average NIR and MIR spectra of thirteen different Zingiberaceae fruits are shown in Figure 4. The average NIR spectra show some similar characteristic peaks at 8330, 6803, 5696, 5179, and 4693 cm^−1^, but there are significant differences in their absorption intensities (Figure 4A). The absorption peak at 8330 cm^−1^ was related to the C-H second overtone stretch in -CH_3_ and -CH_2_; the wave peak at around 6803 cm^−1^ was the first overtone of O-H; the 6000–5400 cm^−1^ region was associated with the first overtone of C-H stretching vibration in -CH_2_; the combination of O-H and C-O stretching resulted in a peak at 5179 cm^−1^ [37]; and the peak at around 4693 cm^−1^ was related to combination bands involving C–H, C=C, and C=O stretching vibrations of lipids, phenols, and other aromatic groups [38].

The MIR spectra of different Zingiberaceae fruits showed a total of nine obvious common absorption peaks at 3289, 2921, 2854, 1623, 1425, 1369, 1315, 1240, and 1010 cm^−1^ (Figure 4B). The wide absorption peak around 3289 cm^−1^ was due to the stretching vibration of O-H contributed by saccharides, glycosides, and water molecules. The absorption peak at 2921 cm^−1^ was related to the C-H asymmetric stretching vibration of -CH_2_ and -CH_3_ [39]. The 1800–1500 cm^−1^ region was associated with C=O stretching vibration, C=C stretching vibration, asymmetric stretching vibration of the carboxyl group involved in the hydrogen bonds, and hydrogen-bond scissoring vibration of the free carboxyl group in pectin, fatty acids, flavonoids, saccharides, and steroid saponin. The 1500–650 cm^−1^ region as the MIR fingerprint region could reflect the subtle changes in molecular structure, and the vibration types in this region were complex and overlapping, but they were highly sensitive to changes in molecular structure, which could be used to distinguish small differences in the structure of different compounds. The absorption peaks at 1425 and 1369 cm^−1^ were related to C-H stretching vibration and bending vibration of -CH_2_ and -CH_3_. The highest absorption peak appeared in the 1200–950 cm^−1^ region, which was related to the C-C stretching vibration, C-O stretching vibration, and C-O-H bending vibration [40].

Using NIR and MIR spectra data, it can be seen that the spectra of these thirteen Zingiberaceae spices show similar trends, which is because there are similar functional groups between the samples, which indirectly indicates the existence of similar metabolic components. However, there are also significant differences in some absorption peaks and intensities, indicating significant differences in compounds between different species, as confirmed by GC–MS data results. Therefore, we can classify different species based on the significant differences in this part.

### 3.3. Comparison of Low-Level and Mid-Level Data Fusion Models

**Figure 5:**
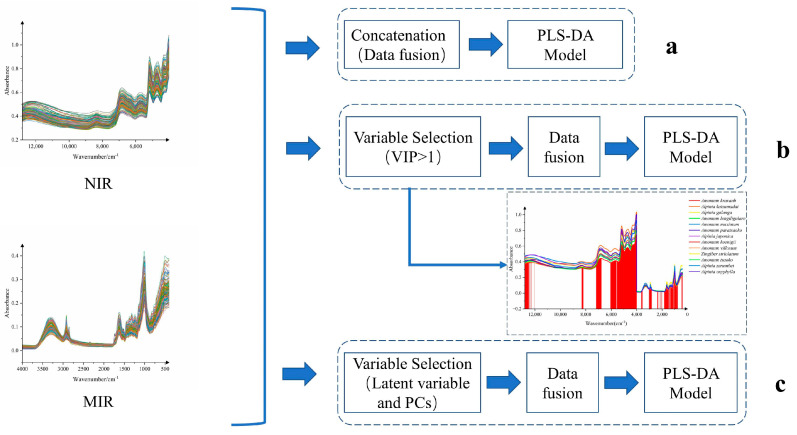
The flow chart of the data fusion processes: (**a**) low-level data fusion; (**b**) mid-level data fusion (VIP > 1); (**c**) mid-level data fusion (latent variable and PCs).

**Figure 6:**
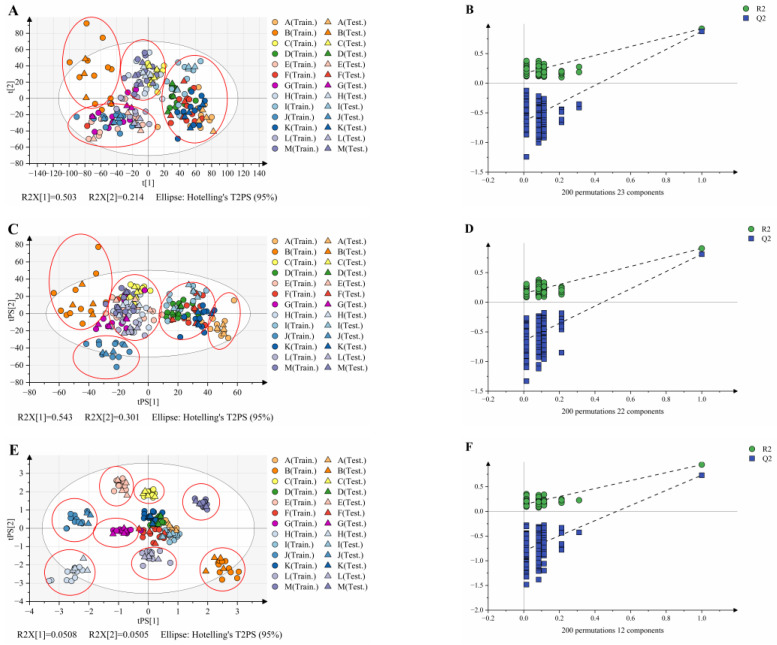
Score plots obtained by PLS-DA of the different data fusions: (**A**) low-level data fusion; (**C**) mid-level data fusion (VIP > 1); (**E**) mid-level data fusion (latent variable and PCs); permutation plot at 200 times of permutations: (**B**) low-level data fusion; (**D**) mid-level data fusion (VIP > 1); (**F**) mid-level data fusion (latent variable and PCs).

Data fusion of the raw data was performed using the method shown in Figure 5, and PLS-DA models were established separately (Figure 6). It could be seen that there was a clustering trend between the different samples from the score scatter plots of the three models. As shown in the red circle in the figure, the scatter plot of PLS-DA for low-level data fusion is roughly clustered into four parts, the scatter plot of PLS-DA for the mid-level data fusion (VIP > 1) is roughly clustered into five parts, while the scatter plot of PLS-DA for the mid-level data fusion (latent variable and PCs) has the best clustering effect, basically clustering thirteen spices. This indicates that the mid-level data fusion models had better clustering performance than the low-level data fusion model. The reason was that the mid-level data fusion performed feature selection, reducing the interference of redundant information, and thereby improving the classification performance of the model. Moreover, the results of the permutation testing (n = 200) of the three PLS-DA models show that all models had no risk of overfitting. The PLS-DA score plot of the mid-level data fusion model for latent variables clusters thirteen species of Zingiberaceae more intuitively and effectively, but its score plot only explained 10.13% of the variation (PC1:0.0508, PC2:0.0505), which indicates that the interpretation ability of the model may be reduced while improving the classification performance of the model by reducing redundant information. Comparing the score plots of these three models, it can be found that the mid-level data fusion model (VIP > 1) has the best performance and classification trend, with PC1 (54.3%) and PC2 (30.1%) explained by 84.4% of the variation.

Although some species were not separated in the score plots, the accuracy of both the training and test sets of the models was 100% (Table 2). The goodness of fit of R^2^Y was more than 90.7%, and the goodness of prediction Q^2^ was more than 83.3%, indicating that the performances of both low-level and mid-level data fusion models were excellent. The mid-level data fusion model based on latent variables has the best performance among them (R^2^Y = 0.952, Q^2^ = 0.913, RMSEE = 0.0121, RMSECV = 0.0450, RMSEP = 0.0122). The results showed that NIR and MIR fusion spectra can effectively identify and classify fruits of different Zingiberaceae species as a non-destructive method.

## 4. Conclusions

This study used GC–MS combined with spectroscopy of NIR and MIR to qualitatively and quantitatively analyze the metabolites of thirteen Zingiberaceae spices and classify them. The results showed that the composition of metabolites from different species can be clearly obtained through GC–MS analysis. A total of 81 metabolites were successfully obtained from 13 spices, with the main volatile metabolites being terpenoids, accounting for approximately 42.55% to 66.67%. Moreover, there were significant differences in the metabolic components and relative contents of different species. Thirty-seven differential metabolites were screened through VIP ≥ 1, with terpenoids and esters accounting for the largest proportion. The classification and discrimination performance of the OPLS-DA model established using GC–MS data is relatively poor. Further, spectroscopy can quickly, simply, and non-destructively classify different Zingiberaceae spices. The different species of Zingiberaceae had similar absorption peaks, but there were significant differences in the intensity of absorption peaks. All PLS-DA models established based on combined spectroscopy had excellent classification performance, among which the mid-level data fusion model based on latent variables had the best performance. In summary, GC–MS and spectroscopy have their own advantages, and their combination with PLS-DA can serve as a powerful tool for classifying different Zingiberaceae species, providing assistance in identifying confusion and adulteration in the market.

## Figures and Tables

**Table 1:** Summary of main metabolites in thirteen Zingiberaceae spices.

Name	Main Volatile Components	Compound Classification	Relative Content * (%)
*Amomum koenigii*	Linalool	Monoterpenes	1.9096 ± 0.1795
	Camphene	Monoterpenes	1.5867 ± 0.5335
	(–)-Camphor	Monoterpenes	1.1778 ± 0.2981
	Borneol	Monoterpenes	1.0792 ± 0.4300
	Limonene	Monoterpenes	1.0783 ± 0.7768
	α-Pinene	Monoterpenes	0.9384 ± 0.1091
*Amomum kravanh*	1,8-Cineole	Monoterpenes	8.6057 ± 2.3572
	β-Selinene	Sesquiterpenes	3.3511 ± 0.2210
	α-Pinene	Monoterpenes	2.6347 ± 0.4049
	(–)-β-Pinene	Monoterpenes	2.3827 ± 1.7027
	Sabinene	Monoterpenes	2.0574 ± 0.2369
	β-Myrcene	Monoterpenes	1.6901 ± 1.2053
*Amomum longiligulare*	Borneol	Monoterpenes	1.4594 ± 1.1597
	Camphene	Monoterpenes	1.4351 ± 0.3747
	β-Myrcene	Monoterpenes	1.2737 ± 0.9165
	Limonene	Monoterpenes	0.9416 ± 1.0558
	Linalool	Monoterpenes	0.8842 ± 0.2345
	Caryophyllene	Sesquiterpenes	0.8372 ± 0.4591
*Amomum tsaoko*	1,8-Cineole	Monoterpenes	8.6216 ± 1.8458
	γ-Muurolene	Sesquiterpenes	3.0686 ± 1.3184
	Limonene	Monoterpenes	2.4858 ± 1.9670
	Linalool	Monoterpenes	2.4640 ± 1.2840
	Copaene	Sesquiterpenes	2.2274 ± 0.6573
	Geraniol	Monoterpenes	1.5468 ± 1.6610
*Amomum villosum*	Linalool	Monoterpenes	2.0851 ± 0.6833
	(–)-β-Pinene	Monoterpenes	1.7271 ± 1.2663
	α-Pinene	Monoterpenes	1.3186 ± 0.3246
	Borneol	Monoterpenes	0.7760 ± 1.0974
	β-Myrcene	Monoterpenes	0.7023 ± 0.9932
	Copaene	Monoterpenes	0.6476 ± 0.9159
*Amomum maximum*	(–)-β-Pinene	Monoterpenes	2.4277 ± 0.1931
	β-Selinene	Sesquiterpenes	1.4536 ± 0.1007
	Copaene	Sesquiterpenes	1.2216 ± 0.0431
	β-Bourbonene	Sesquiterpenes	1.2136 ± 0.1201
	α-Ocimene	Monoterpenes	1.1048 ± 0.7753
	Humulene	Sesquiterpenes	0.9789 ± 0.6923
*Amomum paratsaoko*	(Z)-2-Decenal	Aldehydes	1.4423 ± 0.5351
	Octanal	Aldehydes	1.1835 ± 0.6240
	(E)-2-Octenal	Aldehydes	1.0320 ± 0.4831
	Limonene	Monoterpenes	0.9243 ± 0.2577
	α-Pinene	Monoterpenes	0.8682 ± 0.0838
	α-Ocimene	Monoterpenes	0.7899 ± 0.1907
*Alpinia galanga*	Caryophyllene	Sesquiterpenes	2.1802 ± 1.5630
	Tridecane	Hydrocarbons	1.9142 ± 0.3532
	Decyl acetate	Esters	1.5508 ± 0.5689
	Copaene	Sesquiterpenes	1.3953 ± 0.1010
	Alloaromadendrene	Sesquiterpenes	1.3661 ± 0.0971
	1,8-Cineole	Monoterpenes	1.3611 ± 0.4756
*Alpinia katsumadai*	Estragole	Monoterpenes	1.3972 ± 0.3235
	Acetic acid	Acids	0.2495 ± 0.1786
	(–)-β-Pinene	Monoterpenes	0.2406 ± 0.1470
	o-Cymene	Hydrocarbons	0.2366 ± 0.1923
	1,8-Cineole	Monoterpenes	0.1577 ± 0.0265
	Limonene	Monoterpenes	0.1514 ± 0.0243
*Alpinia zerumbet*	1,8-Cineole	Monoterpenes	2.4631 ± 0.9457
	Linalool	Monoterpenes	2.2433 ± 0.2904
	Limonene	Monoterpenes	1.9982 ± 1.1538
	o-Cymene	Hydrocarbons	1.7801 ± 1.2708
	α-Pinene	Monoterpenes	1.4746 ± 0.2429
	Borneol	Monoterpenes	1.4343 ± 1.0396
*Alpinia japonica*	1,8-Cineole	Monoterpenes	1.8123 ± 0.1452
	Methyl hexanoate	Esters	0.7849 ± 0.1505
	Linalool	Monoterpenes	0.6186 ± 0.1141
	o-Cymene	Hydrocarbons	0.5333 ± 0.0981
	α-Terpineol	Monoterpenes	0.4864 ± 0.0370
	(–)-β-Pinene	Monoterpenes	0.4055 ± 0.0520
*Alpinia oxyphylla*	Citral	Monoterpenes	3.1651 ± 2.6423
	Humulene	Sesquiterpenes	1.6426 ± 1.1672
	(–)-β-Pinene	Monoterpenes	1.5048 ± 0.7049
	α-Ocimene	Monoterpenes	1.4872 ± 0.8404
	Linalool	Monoterpenes	1.4704 ± 0.1282
	β-Myrcene	Monoterpenes	1.4372 ± 0.1290
*Zingiber striolatum*	Linalool	Monoterpenes	0.6799 ± 0.3538
	(–)-Bornyl acetate	Monoterpenes	0.5004 ± 0.0663
	Camphene	Monoterpenes	0.3450 ± 0.1443
	Acetic acid	Acids	0.2345 ± 0.0430
	1,8-Cineole	Monoterpenes	0.2029 ± 0.0813
	Copaene	Sesquiterpenes	0.1585 ± 0.0688

* The relative content was expressed as the average ± standard deviation of three measurements.

**Table 2:** Parameters of PLS-DA models with different data fusion.

Model	Data Fusion Strategy	LV	R^2^Y	Q^2^	RMSEE	RMSECV	RMSEP	Accuracy of Training Set (%)	Accuracy of Test Set (%)
Model I	Low-level data fusion	23	0.921	0.847	0.0789	0.1366	0.0859	100	100
Model II	Mid-level data fusion (VIP > 1)	22	0.907	0.833	0.0836	0.1264	0.0794	100	100
Model III	Mid-level data fusion (latent variables)	12	0.952	0.913	0.0121	0.0450	0.0122	100	100

## Data Availability

Data from the present study are available upon request from the corresponding author.

## Supplementary Materials

The following supporting information can be downloaded at: https://www.mdpi.com/article/10.3390/foods12203714/s1, Table S1: Summary of metabolites of thirteen Zingiberaceae spices.
